# Interpretable prediction of neonatal mortality and its key predictors using machine learning and SHAP analysis

**DOI:** 10.1186/s12911-026-03567-1

**Published:** 2026-05-21

**Authors:** Kerebih Getinet Bitew, Yaregal Assabie, Tesfa Tegegne

**Affiliations:** 1 Faculty of Computing, Bahir Dar Institute of Technology, Bahir Dar University, P.O. Box 26, Bahir Dar, Ethiopia; 2 Department of Computer Science, College of Natural and Computational Sciences, Addis Ababa University, Addis Ababa, Ethiopia; 3 Department of Computer Science, Debre Markos University, Debre Markos, Ethiopia

**Keywords:** Machine learning, Neonatal mortality, Class balancing, SHAP, Prediction

## Abstract

**Introduction:**

Neonatal mortality is still a global public health concern, especially in lower- and middle-income countries like Ethiopia. Machine learning (ML) has shown excellent performance in healthcare prediction tasks due to its capability of efficiently handling interactions within mixed-type tabular data. Therefore, this study aimed to develop an interpretable model that can predict neonatal death based on the Ethiopian Demographic Health Survey (EDHS) dataset.

**Methods:**

This study utilized the imbalanced EDHS dataset collected from all surveys conducted in 2000, 2005, 2011, 2016, and 2019. We evaluated some basic and tree-based ensemble ML algorithms validated with five-fold cross-validation and 80/20 data splitting. Unbalanced, weighted, and SMOTENC class balancing techniques are utilized across both evaluation strategies. The models were compared based on sensitivity and SHAP interpretability with consideration of F1-score and AUC-PR trade-offs.

**Results:**

The weighted LightGBM model achieved the highest recall performance (recall = 87.2%) with a slight reduction of F1 = 85.3% and PR-AUC = 92.6% by preserving SHAP interpretability. The SHAP analyses indicate that breastfeeding initiation, number of living children, and ANC visits are the top three key predictors. The dependence plot shows positive SHAP values for delayed breastfeeding initiation, no living children, no antenatal care, lower household member size, grand multiparity, male sex, small birth size, twin births, and short preceding birth intervals.

**Conclusion:**

The weighted LightGBM model not only had better sensitivity with competitive AUC-PR results but also showed reliable interpretability using SHAP analyses. The model predicted strong neonatal mortality odds associated with delayed breastfeeding initiation, absence of living children, and lack of antenatal care visits. The model also predicted that neonatal mortality would be more likely for low household member size, grand multiparity, male sex, small birth size, twin births, and short preceding birth intervals. Finally, adequate ANC visits and early initiation of breastfeeding were protective and affected survival outcomes for neonates, emphasizing the importance of maternal and newborn healthcare programs.

**Supplementary Information:**

The online version contains supplementary material available at 10.1186/s12911-026-03567-1.

## Introduction

Neonatal mortality is still a global public health concern, especially in lower- and middle-income countries with limited access to high quality maternal and neonatal services [1]. While there has been considerable progress toward Sustainable Development Goals (SDG 3.2), Sub-Saharan Africa continues to experience unacceptably high neonatal mortality rates, and Ethiopia is among countries that face monumental challenges [2, 3]. The findings of the Ethiopian Demographic and Health Survey (EDHS) indicate that deaths of newborns contribute to a large part of the mortality of children under five years, thus pointing out the urgent requirement for data-guided interventions [4, 5].

Prior studies that use DHS data have identified significant risk factors associated with neonatal mortality, including maternal age, parity, prenatal care, and delivery conditions [5, 6]. In addition, many of these studies have given us valuable information about the different types of variables that can help explain neonatal death through the use of multilevel regression and causal approaches, as well as survival analysis methods. Machine learning models, in contrast to statistical techniques that specify a predetermined functional form, can be constructed without having to predetermine what the actual model form will be, which provides much more flexibility in modeling nonlinear relationships and interactions between variables [7, 8]. The developments of ensemble learning—such as Extreme Gradient Boosting (XGBoost), Light Gradient Boosting Machine (LightGBM), and Categorical Gradient Boosting (CatBoost)—have shown excellent performance in healthcare classification tasks due to their capability of efficiently handling high-dimensional and mixed-type data [9, 10].

Tabular data, data structured in rows and columns, is still the most common type of data in practice. These datasets typically involve a combination of numerical and categorical features - a reason for the nature of complexity in feature selection. Tabular data are dominated by multicollinearity among numeric variables [11, 12], high-cardinality categorical attributes [13], skewed distributions [14], and heterogeneity in scales and types of attributes [15]. Besides, relationships between features and the target variable can be non-linear or are intricate ones that cannot be described by standard univariate techniques [16]. These characteristics necessitate the use of robust and flexible feature selection techniques appropriate for tabular data nature. Moreover, there are methodological complexities that arise when conducting ML on EDHS data, including class imbalance (the rarity of neonatal deaths) and the mixed nature of data (categorical and numeric) [17–19].

This study is guided by the proximate determinants and socio-ecological framework to investigate the multiple factors that cause neonatal mortality [20, 21]. The reproductive and biological characteristics of mothers, which include their age and parity and birth spacing, determine neonatal survival because these factors create obstetric hazards and lead to premature births and low birth weight babies [22]. Household size and household wealth determine access to nutrition and caregiving capability as well as the use of health care, which means both indirectly influence rates of neonatal mortality [23]. Health services such as antenatal care and skilled delivery help decrease rates of neonatal mortality through early diagnosis and identification and treatment of complications related to delivery and pregnancy [24, 25]. Neonatal morbidity and mortality rates also correlate with the environment and context in which people live, such as their region and climate zone. These factors impact both the ecology of the disease and how much access to a healthcare system someone has [26]. To use this multilevel framework, ML methods will be used to capture the complexity and nonlinearity of the relationships between all of these variables and their respective impacts [27].

SHAP (SHapley Additive exPlanations) assesses the impact of features by providing a mathematically consistent measure of the marginal effect of each variable on the predictions of the model. Recently, however, researchers have been cautioned against drawing overly strong conclusions regarding post-hoc explanation methods based on the results of machine learning applied to health-related data [28–30]. The importance scores calculated by using machine learning (including SHAP) to explain the health data do not necessarily yield causal conclusions about features being attributable. Rather, they can potentially reflect confounding, mediation, feature correlation, or proxy relationships present in the health data, and therefore these scores are highly sensitive to how the model is specified (and trained) [30, 31]. The important features in this paper follow the guidance described above. Thus, they are used only to provide transparency, identify key predictors of risk, and develop future testable hypotheses regarding causality related to the health data, but not to determine the underlying causal effect.

This study aims to contribute, in three distinct ways, to the development of an interpretable neonatal mortality prediction model from DHS data for lower- and middle-income countries. First, we present a systematic evaluation of three different imbalance-handling methods, which include unbalanced, weighted, and SMOTENC-based approaches across various ML models, because we aim to show how these methods affect performance and robustness, which remain hidden in current DHS-based neonatal mortality research. Second, we assess the performance of tree-based ensemble models together with basic statistical models by applying the same preprocessing and validation methods, which enables us to compare their performance improvements against established models. Third, we conducted SHAP analysis to determine which predictors significantly contribute to the best-performing combination of imbalance-handling technique and prediction model.

## Methods

### Study area

This research relied on data collected from the Ethiopian Demographic and Health Survey (EDHS), which is a survey representing the whole country and including all areas and cities of Ethiopia. The survey represents both urban and rural populations and uses a stratified two-stage cluster sampling technique that is based on census enumeration areas.

### Study period and study design

This research utilized secondary data from five different rounds of EDHS with repeated cross-sectional design (i.e., the 2000, 2005, 2011, 2016, and 2019 EDHS).

### Study population

The study population included live births reported in the birth histories of women aged 15 to 49 years. Each survey collects data about the last five years preceding it.

### Data source

The Ethiopia Demographic and Health Survey (EDHS) data collected from the first to the most recent survey in 2000, 2005, 2011, 2016, and 2019 was used in this study. Each round of the EDHS is an independent, nationally representative, cross-sectional survey that does not intentionally include any of the same households as previous rounds. These dataset was collected by the Central Statistical Agency and the Ethiopian Public Health Institute in support of the Demographic and Health Surveys Program (4). After requesting it officially, the dataset was accessed through the DHS Program database via the link https://dhsprogram.com/data/available-datasets.cfm.

### Study variables

The **dependent variable**, Neonatal_Dead, was created from child alive data in the KR files. A child was classified as “Yes” (neonatal death) if they died within the first month of life or “No” (survived) if the child survived until the interview and was aged one month or older. Records of alive neonates younger than one month of age were excluded because they have a chance of death until they turn a month.

**Predictor variables** included maternal characteristics (age, education, age at first birth, marital status, contraceptive method used, parity, religion), reproductive and health service factors (Antenatal Care(ANC) visits, tetanus vaccination, place of delivery, birth order), child characteristics (sex, twin birth, breastfeeding initiation, birth size, preceding birth interval), and household and environmental factors (wealth index, household size, water source type, region, residence type, climate zone, year). These were chosen based on evidence and theory related to neonatal survival. See definition of variables in supplementary file S1.

### Inclusion and exclusion criteria

The study included only live-born neonates for the last five years before the survey who had enough available retrospective information to ascertain their neonatal survival status. However, the study excluded stillbirths and live births that lacked clear information about neonatal survival. Additionally, we excluded alive neonates who had not completed 28 days of neonatal age at the time of data collection to avoid misclassification of dead neonates shortly after the survey.

### Modeling framework

**Fig. 1:**
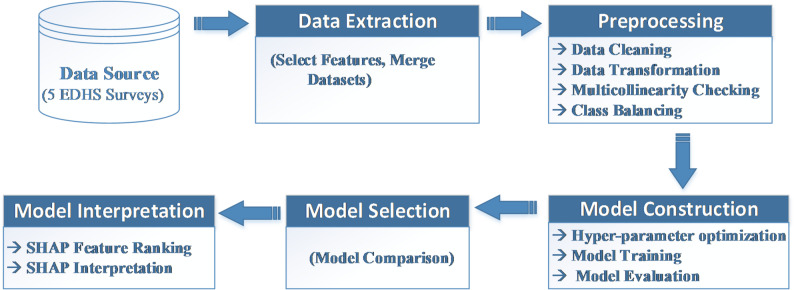
Model framework diagram

The workflow for data preprocessing and model building contains five step-wise stages as shown in Fig. 1. These are data extraction, preprocessing, model construction, model selection, and model interpretation.

### Data extraction

In this study, data were extracted from the children’s recode (KR) files from each round of the survey and pooled together, all of which contain information regarding live births in the five years before each survey. The combined dataset contained a total of 40,680 participants, among which 38,899 (95.6%) were living and 1,781 (4.4%) died in the neonatal period (0–28 days). The dataset provides a wide range of information on maternal, child, and household characteristics, as well as health service utilization, and neonatal outcomes.

### Preprocessing

#### Data cleaning

Data cleaning steps included string normalization, label harmonization, handling of missing data, and constructing standardized variables. Categorical features were normalized through the correction of spelling variations, inconsistencies in case, and labels that have the same meaning (such as the ‘Muslim’, ‘Moslim’, and ‘Islam’ values of religion) and then consolidated into unified categories, thus avoiding the creation of an artificial category split of one category into multiple categories. Non-standard forms of missing data (i.e., blank strings, “NA,” and “don’t know”) were recoded into consistent representations. Label encoding was utilized on the existing observed categories prior to the imputation of missing values, after which the imputed values were decoded back to their original categorical labels to maintain multivariate dependencies. Furthermore, imputations were finalized prior to any sampling was performed to ensure the complete distributional structure of the data was used to estimate missing values to avoid any possible bias from performing imputations on data that was reduced.

The dataset consists of variables with missing values such as birth size (13.09%), drinking water source type (1.85%), wealth index (0.65%), birth interval (0.15%), and religion (0.01%). To solve this problem, a KNN imputation method with k = 5, which used Hamming distance to measure the similarity among categorical records, was employed. The KNN approach was selected because it maintains inter-variable relationships and retains categorical data properties in complex survey datasets [32, 33]. The key predictors were harmonized according to the DHS and WHO definitions to ensure comparability across the different EDHS survey waves (2000–2019), including climate zones [34], cooking fuel, drinking water, tetanus toxoid vaccination, antenatal care visits, birth order, and breastfeeding initiation timing [35, 36]. The descriptive summary of variables were shown in supplementary file S1 Tables 1, 2 and 3.

#### Data transformation

Recoding on some of the variables were done prior to model training to guarantee analytical consistency, enhance model interpretability, and minimize sparsity. Label encoding of categorical variables was used with respect to tree-based models. As a result, encoded integers act only as category identifiers and have no numerical value. When building decision trees, threshold rules are applied to make splits to define the categories; linear distance between the encoded values does not provide any correlation for decision trees. Therefore, the labeled values do not impose an ordinal-type structure on the data, like linear models.

On the other hand, one-hot encoding was used for linear models and distance-based models such as LR and KNN, ensuring that all category levels became independent features, thus preventing manipulation of bogus ordinal relationships. The final dataset created through a model-appropriate encoding method and sound treatment of missing data and multicollinearity was standardized, consistent, and suitable for modeling and later feature selection.

#### Multicollinearity checking

Evaluating multicollinearity is crucial for establishing the independence of predictors to avoid redundancy. In this regard, a mixed-type association analysis, which can handle categorical and numerical variables, was implemented to evaluate whether multicollinearity exists. To investigate the relationship between the variables, a mixed-type association heatmap was created using Cramer’s V to assess categorical–categorical pairs, correlation ratio (η²) for numeric–categorical pairs, and Spearman’s ρ for numeric–numeric pairs [37].

**Fig. 2:**
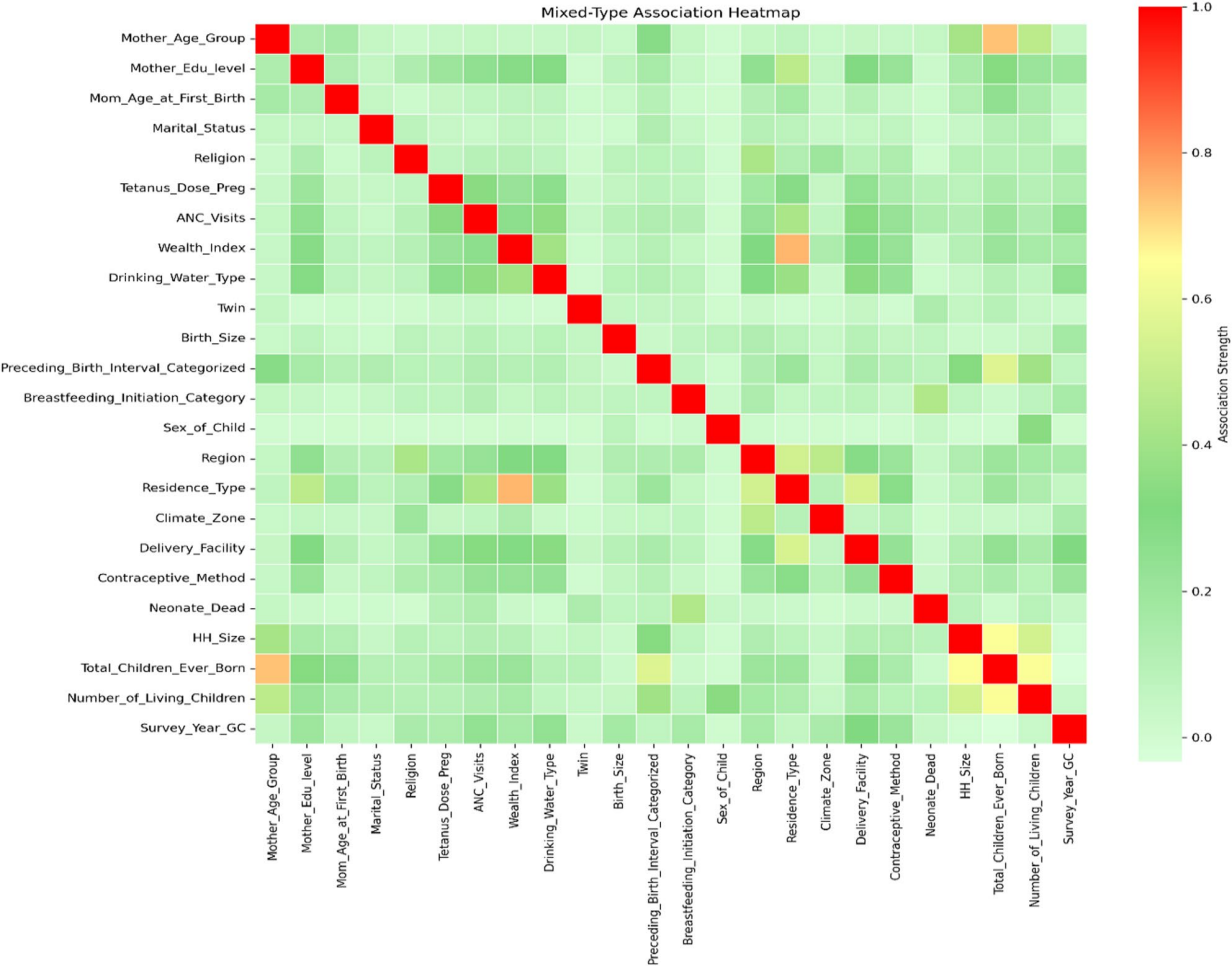
Multicollinearity of features based on mixed-type association analysis method

The association matrix depicted in Fig. 2 indicates that the majority of variable pairings exhibited weak to moderate associations which is less than < 0.8 correlation coefficients [37, 38], signifying minimal redundancy among most predictors and affirming a suitable degree of independence in the data for multivariate modeling.

#### Class balancing

We utilized a structured multi-strategy approach to manage imbalance handling. Firstly, in an effort to minimize the drawbacks of extreme data imbalance (≈ 96:4 alive-to-death ratio), under-sampling to a moderate level was conducted using year and region as stratification variables to maintain both temporal and spatial representativeness [39, 40]. We utilized two controls for each case, i.e., a majority-to-minority ratio of 2:1, to minimize the predominant exposure but still provide enough data diversity to facilitate accurate learning [41]. This stratified undersampling leads to better stability, which decreases the chances of producing unrealistic synthetic observations when SMOTENC is applied [42]. Following the stratified undersampling, four learning strategies were evaluated to the new dataset: unbalanced baseline (the stratified dataset with no further adjustments), cost-sensitive learning (class weighting), synthetic minority oversampling (using SMOTENC), and five-fold CV. These strategies were compared as being mutually exclusive, allowing for an empirical analysis of the trade-offs associated with each.

SMOTENC, an extension of a classical SMOTE to mixed types of data [18], was chosen as the candidate imbalance-handling method because the DHS dataset includes both categorical and numeric predictor variables, and SMOTENC enables synthetic oversampling without a complete one-hot encoding of its categorical features. The use of the method formed part of an overall framework that maintained the integrity of the survey design throughout different times. Temporal pooling across DHS waves was regarded as a series of independent cross-sectional samples instead of a longitudinal process. In this context, SMOTENC functions locally within feature spaces to enhance minority-class representation for prediction, without modifying the distribution of validation or test data [43, 44]. In the study, SMOTENC was applied only on the training dataset to prevent data leakage during the model building. When it comes to the validation and test set, they are used with their original stratified survey distribution and with no synthetic observations. Therefore, the reported performance metrics can only reflect the generalization of the model to non-synthetic data, thus minimizing the effect of any resampling distorting the distribution from the original in evaluating the performance of the model. Consequently, SMOTENC is employed to improve minority-class differentiation within a predictive framework, rather than to restore population-representative joint distributions.

Additionally, weighted learning, which is an algorithmic-based class balancing method, was also used. Weighted learning fused greater misclassification costs for the minority-class observations by implementing class weights inversely proportional to the observed class frequencies when training the model [40]. The unbalanced baseline was used as a comparison with the balancing strategies to assess their effect on both recall and discrimination performance.

### Model construction

Model selection was primarily guided by performance for the minority class and its SHAP interpretability. Robustness—consistent high performance across strategies—and stability—lowest variability across folds—were also assessed. The evaluation of model performance was predominantly conducted by 5-fold cross-validation to achieve stable and reduced-variance estimates for the purposes of model comparison and selection. Furthermore, an 80/20 train–test data splitting validation strategy was also conducted as an independent hold-out evaluation to verify the generalization performance on unseen data. The robustness and stability of the models were assessed based on the consistency of five-fold CV and 80/20 train-to-test data splitting evaluation strategies.

#### Hyper-parameter optimization

The Optuna framework was utilized for hyperparameter optimization in all evaluated models. It employs Bayesian optimization and sequential model-based search, which are efficient methods to search through the parameter spaces [45]. The parameter sets with an 80/20 stratified hold-out evaluation that achieved the highest recall, shown in supplementary file S1 Table 4, were retained and then used for model evaluation.

#### Model training

A variety of classification algorithms were applied, including the traditional algorithms of DT, LR, and KNN, in addition to the ensemble tree-based models of RF, GBM, XGBoost, LightGBM, and CatBoost. Ensemble-based algorithms were considered due to their ability to model nonlinear interactions and variable relationships that were heterogeneous [18]. However, basic models were included as baseline approaches for comparison purposes with the more complex models due to their large use in epidemiological studies and easily interpretable parameterization.

Models were trained and evaluated under three different class balancing strategies: (a) unbalanced learning with no modifications to the under-sampled dataset used as is; (b) class-weighted learning that assigns more weight to misclassifications of the minority class; (c) oversampling the training dataset using SMOTENC. Five-fold CV and 80/20 hold-out validation were applied to each balancing strategy.

#### Model evaluation

Model performance was evaluated using recall, F1-score, and PR-AUC, which are all metrics particularly fitting for imbalanced classification problems. Recall (sensitivity) measures the percentage of neonatal death cases that were correctly identified, F1-score combines precision and recall to represent the overall classification accuracy, and PR-AUC shows the trade-off between precision and recall over different thresholds [46, 47]. These metrics were prioritized because, from a clinical and public health perspective, accurately identifying these deaths is more important than overall accuracy of the model, given the high cost of false negatives in mortality prediction situations. For completeness, we also presented heatmap plot for Accuracy, Precision, and ROC-AUC evaluation metrics.

### Model selection

Model selection was primarily guided by performance for the minority class and its SHAP interpretability. Robustness—consistent high performance across strategies—and stability—lowest variability across folds—were also assessed. The evaluation of model performance was predominantly conducted by 5-fold cross-validation to achieve stable and reduced-variance estimates for the purposes of model comparison and selection. Furthermore, an 80/20 train–test data splitting validation strategy was also conducted as an independent hold-out evaluation to verify the generalization performance on unseen data. The robustness and stability of the models were assessed based on the consistency of five-fold CV and 80/20 train-to-test data splitting evaluation strategies.

### Model interpretation

#### SHAP feature ranking

In this study, the global feature importance was established by means of a ranking based on the mean absolute SHAP values, while beeswarm and dependence plots were created to show the direction and extent to which specific feature values raised or lowered the predicted mortality risk. All SHAP-based interpretations provide descriptive summaries of model behavior, which depend on the selected features, encoding methods, and techniques for handling imbalances. However, they do not indicate causation. The SHAP patterns for label-encoded categorical predictors show learned decision rules instead of demonstrating ordinal relationships.

#### SHAP interpretation

The chosen predictive model was made transparent through SHAP, which was used to interpret it. Synthetic samples generated by SMOTENC can modify the shape of the original data distribution—introducing variance and boundary distortion [40]—possibly leading to bias in estimating SHAP values [18, 48]; therefore, we conducted our SHAP analysis on models trained with unbalanced or class-weighted datasets to not bias our interpretations of the relationships in the real world. Explainability analysis was restricted to the best model according to the selection criterion and compared to the top-performing models of each strategy combination to ensure consistency.

### Reproducibility

All preprocessing, modeling, and evaluation procedures were implemented in Python 3.10 using the scikit-learn (v1.4.2), imbalanced-learn (v0.12.3) and pandas (v2.2.2) libraries. The ensemble models were implemented through its respective open-source package, XGBoost (v2.1.1), LightGBM (v4.3.0) and CatBoost (v1.2.5), supporting all computations are fully reproducible in the workflow.

## Results

### Model performances

#### Model performances of learning strategies with a 80/20 hold-out validation

**Fig. 3:**
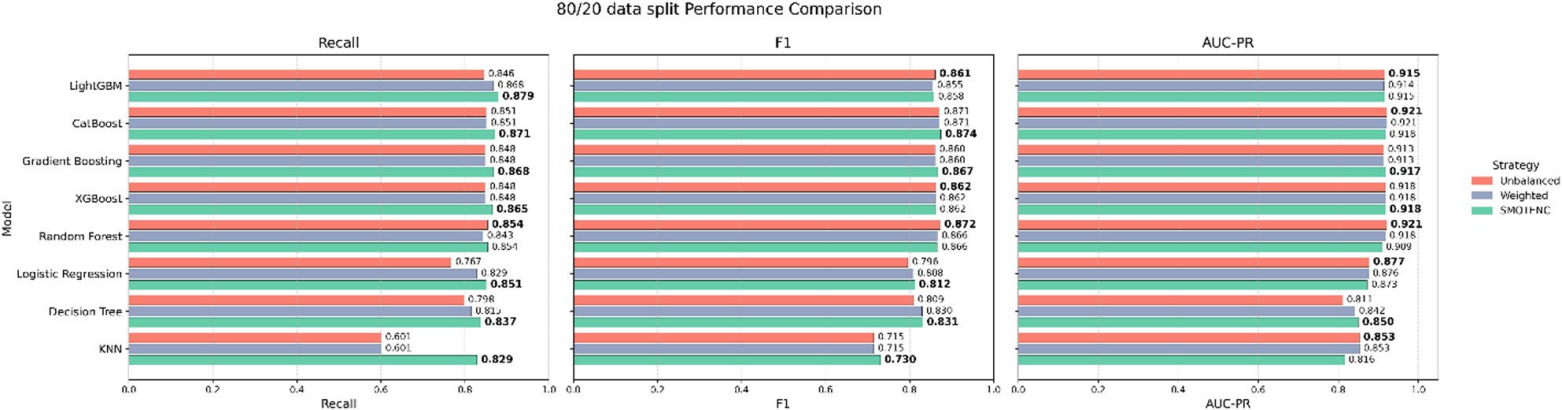
Comparison bar plots model performances across class balancing strategies evaluated using 80/20 hold-out validation

In the unbalanced learning with a hold-out validation, ensemble models outperformed traditional classifiers, as shown in Fig. 3. High-performing models with good trade-offs between precision and recall as well as high discriminative ability include Random Forest (recall 85.4%, F1 87.2%, PR_AUC 92.1%, precision 89.1%), CatBoost (recall 85.1%, F1 87.1%, PR_AUC 92.5%, precision 89.1%) and XGBoost (recall 84.8%, F1 86.2%, PR_AUC 92.7%). GBM (recall = 84.8%) and LightGBM (recall = 84.6%) also maintained high F1, PR-AUC, and precision with scores greater than 86%, 91%, and 87%, respectively. The simpler models displayed lower recall (60.1%, 76.7%, and 79.8% for KNN, LR, and DT, respectively) and low F1 score (ranging from 71.5% to 80.9%) regarding the imbalanced data. (See supplementary file S2 Fig. 1)

The performance of models trained under a weighted learning approach is illustrated in supplementary file S2 Fig. 1, where the LightGBM model achieved a recall of 86.8%, CatBoost a recall of 85.1%, and XGBoost a recall of 84.8%. All of the tested ensemble algorithms were also able to achieve higher recall, F1, PR-AUC, and precision ranging from 84.3% to 86.8%, 85.5% to 87.1%, 91.3% to 92.1%, and 84.2% to 89.1%. However, LR had a competitive recall (82.9%) from the basic models but a lower F1 (80.8%) and PR-AUC score (87.6%), which implies lower precision (78.9%). KNN had the worst recall (60.1%) and was associated with lower F1, PR-AUC, and precision values (71.5%, 85.3%, and 88.1%, respectively), indicating limited adaptability to class imbalance even with the weighting.

Furthermore, performance of the models was computed after implementing SMOTENC on the training set, followed by an 80/20 split of the subsequent data. As shown in supplementary file S2 Fig. 1, LightGBM performed the highest recall with 87.9%, along with 85.8% F1, 91.5% PR-AUC, and 83.7% precision score. CatBoost achieves the best balance between recall and precision, with a performance of 87.1% recall, 87.4% F1, 91.8% PR-AUC, and 87.8% precision. The performance of traditional classifiers is inferior to that of ensemble methods, namely LR (85.1% recall, 81.2% F1, 87.3% PR-AUC, and 77.7% precision), DT (83.7% recall, 83.1% F1, 85.0% PR-AUC, and 82.5% precision), and KNN (82.9% recall, 73.0% F1, 81.6% PR-AUC, and 65.3% precision).

### Model performances of class balancing strategies 5-fold CV

**Fig. 4:**
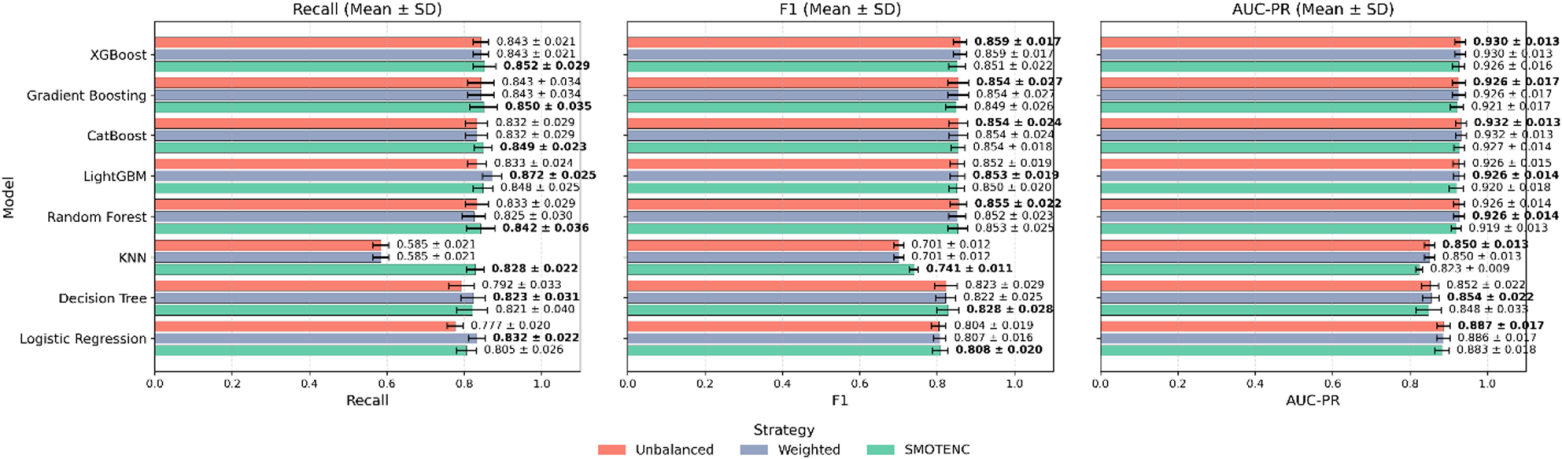
Comparison bar plots model performances across class balancing strategies evaluated using five-fold CV

In a five-fold cross-validation, ensemble models trained with unbalanced data showed nearly the same evaluation metrics values, with ranges of 83.2% to 84.3% recall, 85.2% to 85.5% F1, 92.6% to 93.2% PR-AUC, and 86.5% to 87.9% precision (Fig. 4 and supplementary file S2 Fig. 2). KNN shows the worst performance with 58.5% recall, 70.1% F1, 85% PR-AUC, and 87.6% precision. Similarly, DT and LR are lower performers with 79.2% and 77.7% recall, 82.3% and 80.4% F1, 85.5% and 88.7% PR-AUC, and 85.6% and 83.5%, respectively.

The results of the weighted 5-fold cross-validation modeling confirm that ensemble models, such as LightGBM (recall = 87.2%, F1 = 85.3%, PR-AUC = 92.6%, precision = 83.4%), XGBoost (recall = 84.3%, F1 = 85.9%, PR-AUC = 93.0%, precision = 87.7%), GBM (recall = 84.3%, F1 = 85.4%, PR-AUC = 92.6%, precision = 86.5%), CatBoost (recall = 83.2%, F1 = 85.4%, PR-AUC = 93.2%, precision = 87.8%), and Random Forest (recall = 82.5%, F1 = 85.2%, PR-AUC = 92.6%, precision = 88.2%), perform better than basic classifiers. The KNN has the lowest performance with a recall of 58.5%, an F1 score of 70.1%, a PR-AUC of 85%, and a precision of 87.6%.

Using the SMOTENC balancing strategy with five-fold cross-validation leads to recall rates between 84.2% and 85.2%, F1 scores from 84.9% to 85.4%, PR-AUC values ranging from 91.9% to 92.7%, and precision rates from 84.8% to 86.4%. While KNN achieves 82.8% recall, 74.1% F1 score, 82.3% PR-AUC, and 67% precision, it has the lowest performance among the evaluated models.

### SHAP feature importance

**Fig. 5:**
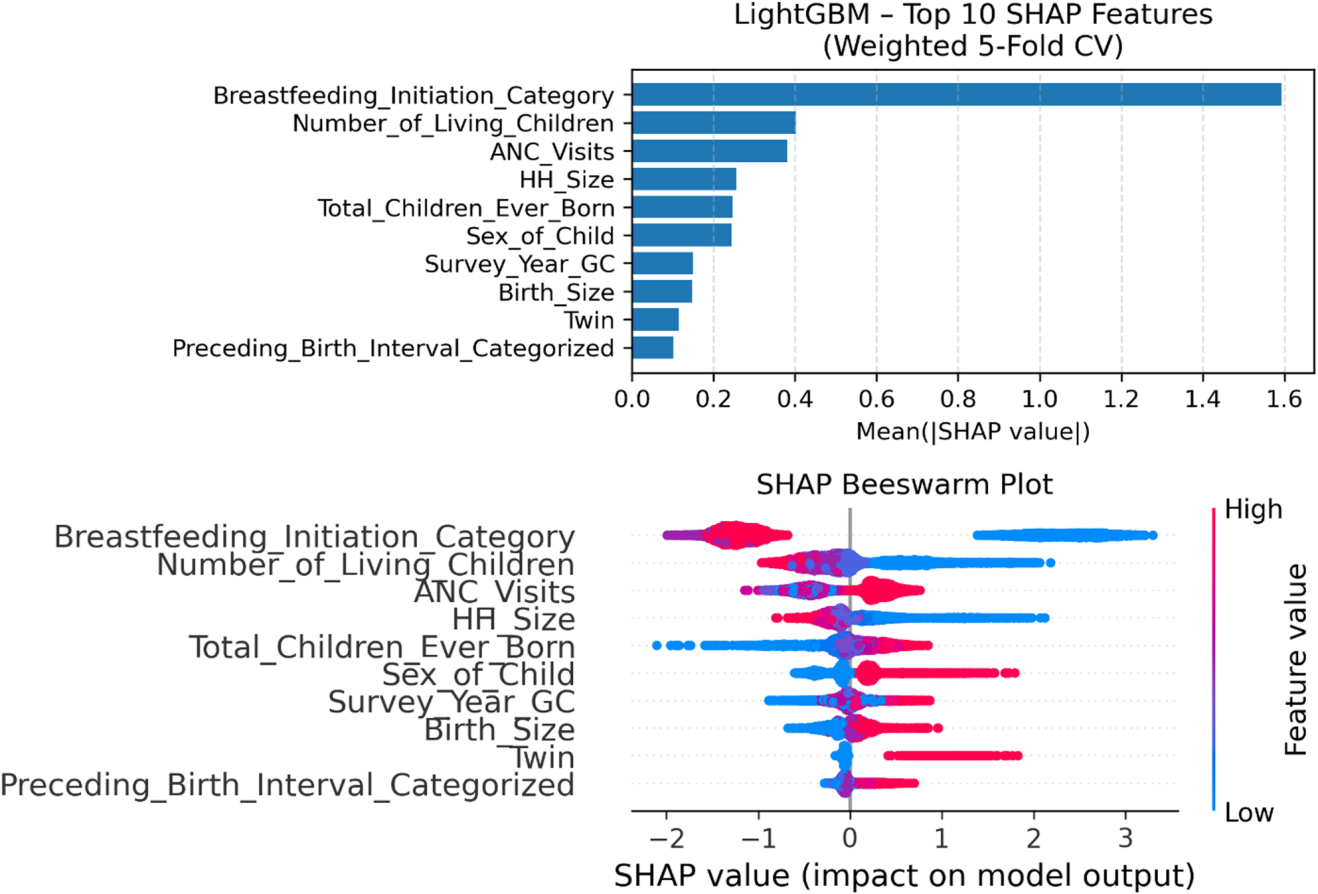
SHAP summary plots for top ten features of weighted LightGBM evaluated using five-fold CV: (**A**) Feature importance bar plot, (**B**) Beeswarm plot

The SHAP summary plot for the weighted LightGBM model, developed using a five-fold cross-validation method (Fig. 5), indicated that the initiation of breastfeeding, number of living children, antenatal care visits, household size, parity, sex, year, birth size of children, twin status, and preceding birth interval are the ten most significant features in predicting neonatal mortality. These were similar as the XGBoost trained using five-fold cross-validation with the unbalanced dataset, as also demonstrated by the SHAP importance correlation (r = 0.995) and the ‘allclose’ test (returns ‘False’). (Supplementary file S2 Fig. 3)

In 80/20 hold-out evaluation, the LightGBM model, trained with a weighted balanced dataset, identifies the top ten predictors as breastfeeding initiation, number of living children, ANC visits, parity, household size, sex, birth size, year, twin status, and preceding birth interval. Similarly, the top ten predictors of the RF model trained on an unbalanced dataset, were breastfeeding initiation, number of living children, ANC visits, household size, sex, parity, twin status, size at birth, mother’s tetanus vaccination during pregnancy, and the preceding birth interval. RF introduces only ‘mother’s tetanus vaccination during pregnancy’ instead of ‘year’ from among top ten predictors when compared with other models. (Supplementary file S2 Figs. 4 and 5)

### SHAP interpretability

**Fig. 6:**
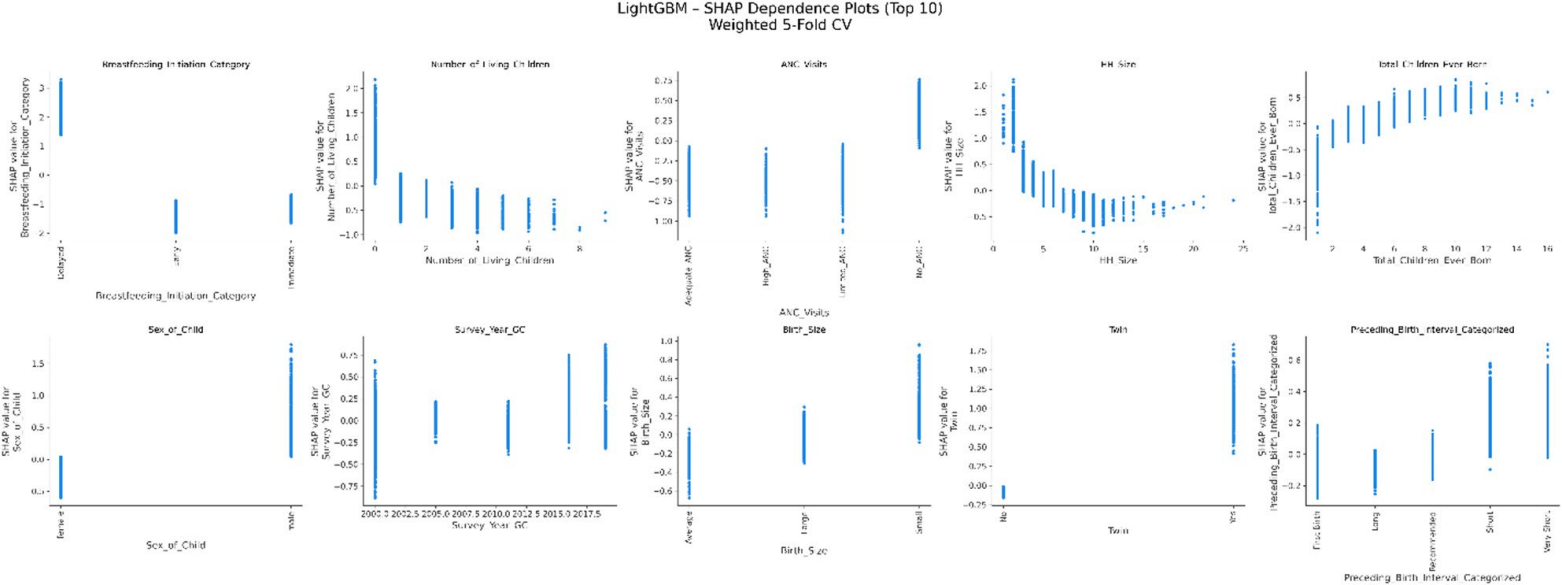
SHAP dependence plots for top ten features of weighted LightGBM evaluated using five-fold CV

The SHAP dependency plots were examined for the top predictors identified in the summary analysis to better understand how individual features influence the model predictions. The figures, as shown in Fig. 6, illustrate the influence of feature values on neonatal mortality predictions, highlighting both nonlinear effects and their interactions with other variables. It indicated an elevated positive SHAP values for delayed breastfeeding initiation, no living children, and no antenatal care. Furthermore, the model assigned a higher positive SHAP values to lower household member size, grand multiparity, male sex, small birth size, twin births, and short preceding birth intervals. On the contrary, both immediate initiation of breastfeeding and adequate ANC visits had high negative SHAP values.

**Fig. 7:**
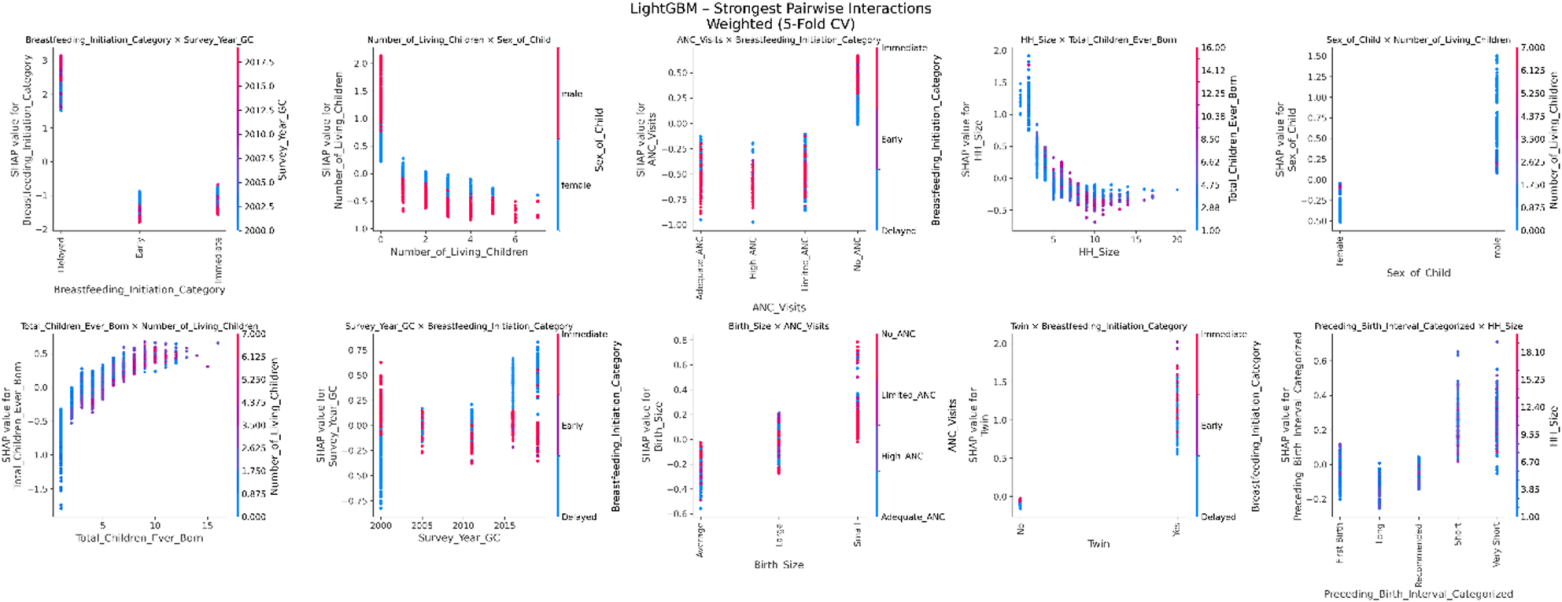
SHAP pairwise dependence plots for strongest pairwise interactions of weighted LightGBM evaluated using five-fold CV

The SHAP pairwise dependence analysis of the weighted LightGBM model, as shown in Fig. 7, found significant non-linear and interaction predictions across the data points. A delay in initiating breastfeeding universally yielded a higher SHAP value for neonatal mortality, while immediate initiation was protective across all survey years. The number of living children followed a non-linear path: mothers that had no prior surviving children had the highest SHAP value, with risk reducing at moderate (1 to 3) parity and increasing very slightly at very high (6+). Predicted risk was higher for male neonates in a low-number-of-living-children household, and significant interaction effects existed between inadequate ANC visits and delayed initiation of breastfeeding, small birth size and no ANC, and birthing twins and delayed initiation—these combinations increased predicted mortality substantially. Short or very short birth intervals prior to the index birth were also related to higher mean SHAP values (particularly for smaller households). Overall, the model identified multiple compounded vulnerabilities instead of isolated linear effects.

## Discussion

### Class balancing and model selection

Class imbalance can significantly lower efficacy in detecting minority classes by biasing toward the majority class [18, 40]. This study shows that the overall effectiveness of class weighting and SMOTENC was model-dependent. The boosting-based models (CatBoost, XGBoost, GBM, and LightGBM) demonstrated low sensitivity to balancing strategies, where the magnitude of the change in both F1-scores and PR-AUC was generally less than 1%. This indicates that the methods of gradient boosting were able to adaptively optimize for moderate class imbalance within the models themselves [9, 10, 49].

Class weighting provides moderate improvements in sensitivity to logistic regression and marginal enhancements to LightGBM and decision trees. On the other hand, Random Forest declines in recall slightly, while some models do not change. The results from LightGBM indicated a better recall of the minority class while using class weights instead of unbalanced ones, with only a slight degradation in PR-AUC. It appears that cost-sensitive learning might be a preferred alternative to balancing strategy in some situations [10, 39].

SMOTENC provides the largest recall values overall; while precision slightly dropped, it was effective in increasing the identification of minority cases of death and has known trade-offs that result from oversampling [18, 40]. The improvement in recall demonstrates that SMOTENC can create synthetic minority class data without losing original data integrity for mixed-type features [42]. It is observed that KNN is substantially increased in recall when SMOTENC is applied; however, KNN has lower precision, indicating an inherent tradeoff between identifying instances within the minority classes versus making incorrect predictions (false positives). These results are consistent with previous work that established that oversampling with synthetic data can increase variance and shift decision boundaries [42, 50].

### Model interpretability and key predictors

This study integrates SHAP with the weighted LightGBM and compares it with models with the highest recall from each strategy combination—unbalanced XGBoost from 5-fold CV and unbalanced RF and weighted LightGBM from 80/20 hold-out validation—to assess its stability. The SHAP summary bar plots—which show the order of feature importance—and dependence plots—which show the effect of feature values on the prediction model—ensure that weighted LightGBM is stable and robust for interpretability analysis. The combined SHAP plots of weighted LightGBM create an interpretability framework that provides a clear understanding of the model. These visual explanations, considered together, provide a confirmation that the model is able to feature established epidemiological relationships and generate clinically and demographically reasonable patterns. The explanations only indicate predictive associations but not causal connections between variables.

According to the SHAP feature importance plot, **breastfeeding initiation timing** is most likely the largest contributor to neonatal death among many possible predictors. The beeswarm and dependence plots further show how delayed initiation will greatly increase the predicted risk of neonatal death, while earlier or immediate initiation would decrease that same predicted risk. The predictive pattern shown here for breastfeeding initiation also supports the well-established epidemiological evidence that indicates early initiation of breastfeeding is linked to greater neonatal survival [51–54]. Studies also show that immediate mother’s breastfeeding initiation after childbirth, within the first hour of life, is consistently associated with improved survival outcomes due to its high nutrition, thermoregulation, immunity benefits, and early mother-child bonding [51, 55–57]. In addition, the model predicts a high risk of mortality for delayed breastfeeding initiation, confirming previous EDHS and WHO research on the issue indicating that early initiation should be considered as a life-saving intervention [4, 58].

The association between the **number of living children** and the risk for neonatal mortality is usually nonlinear. Women with no living children being first-time mothers are at higher risk for neonatal mortality than other women—as supported by positive SHAP scores of the model—because of biological immaturity, obstetric complications, or having little experience caring for young children [59, 60]. Based on the definitions in the DHS, this variable provides a representation of current survival rather than an aggregate measure of fertility exposure; consequently, lower values may also proxy for previous neonatal death. Previous research indicates that child deaths tend to cluster together within high-risk families; therefore, adverse incidents are believed to increase the risk for future neonatal deaths via continued biological, socio-economic, or healthcare-related risk factors [61]. Therefore, the model uses current survival as a proxy risk marker, rather than suggesting a causal relationship between the number of children and the likelihood of their survival. As such, it is a crucial variable to include in any DHS-derived statistical models analyzing neonatal mortality. However, caution should be taken when interpreting this variable; it may also serve as an indicator of survival patterns from previous births, which could introduce reverse causation if not appropriately controlled.

Utilization of maternal healthcare, particularly **ANC visits**, generally has an associational protective predictive effect. Adequate ANC resulted in negative SHAP values (reduction in predicted risk of mortality), while lack of ANC produced a positive effect on predicted risk. The model’s sensitivity to ANC visits is in line with the findings that have shown that early and adequate ANC visits not only help in the detection of high-risk pregnancies but also allow for the application of preventive measures, which ultimately result in reducing mortality [62–66]. The SHAP analysis also shows that a larger **household size** had negative SHAP values; this means those with larger household sizes were protected from neonatal mortality. The findings support previous research from Sub-Saharan Africa, which showed that mothers in big households had lower neonatal death rates than mothers in small households because big households provided more caregiving resources and health access and shared duties among their members [67, 68]. The results are also in line with the household care-support hypothesis, which suggests that extended family systems help reduce neonatal risks by providing improved care and resource distribution to their members [69].

The positive SHAP association between high **total children ever born** (grand multiparity) and predicted mortality risk is consistent with the “Maternal Depletion Theory,” by which mothers lose or deplete nutritional and physiological stores that ultimately impact infant health and neonatal mortality risk by increasing childbearing frequency across their reproductive history [70, 71]. This finding was also consistent with previous observational evidence that suggested high parity had significant associations with higher risk for negative perinatal outcomes, including low birth weight, preterm birth, and neonatal death [72, 73].

**Male** neonates positively contribute to the SHAP value, indicating a higher predicted risk of death. This result is consistent with previous epidemiological studies that compared to females, male neonates have the biological disadvantage in survival—commonly linked to the predictors of slower lung maturation, hormonal differences, and the higher susceptibility to respiratory distress syndrome—which leads to consistently higher neonatal mortality rates in male populations [74–76]. Shifts in neonatal mortality risk were reflected in the predictions made using the survey **years** as a time variable, showing that there are changes to the patterns of risk over the years. The temporal patterns may reflect the improvement in maternal-child health programs—such as health service coverage, skilled birth attendants, and community-based neonatal care—and correspond with national trends to decrease the rate of neonatal mortality [4, 58]. Including temporal features in the model will help broaden its application and decrease the likelihood of it overfitting one survey wave.

Indicators of biological vulnerability also had an important role as predictors of future mortality risk. **Twin births** and small **birth sizes** showed a high SHAP value and indicated a greater mortality risk prediction. These findings are consistent with long-standing clinical evidence showing that multiple gestation pregnancies—more likely to result in premature births, intrauterine growth restriction, and complications during labor and delivery [77, 78]—and low-birth-weight newborns—due to immature organ systems, poor thermoregulation, and increased risk of infection and respiratory problems [79–81]—are significant contributing factors to neonatal mortality. Additionally, short **preceding birth intervals** were positively associated with predicted mortality risk, consistent with demographic hypotheses and maternal depletion hypotheses [82].

The model further detected compounded vulnerability to neonatal death when inadequate ANC co-occurred with delayed initiation of breastfeeding. This compound impact provides evidence of the clinical relevance of integrated maternal and newborn healthcare programs in terms of predicted outcomes. This is because adequate ANC utilization leads to better birth preparedness, which results in improved early postnatal practices through timely breastfeeding initiation that helps increase neonatal survival rates [83–85]. Interactions between short birth intervals and household constraints also suggest vulnerability as captured through the model. Importantly, these results represent associations from the model’s behavior rather than causal impacts. However, the constancy of these interactions through cross-training provides support for the stability and interpretability of the model to identify high-risk newborns.

The identified predictors in the model include structural predictors (parity, total number of live births, household size, and survey year) that tap into demographic transition, socio-economic context, and cohort effects because they are not direct targets for intervention. Previous demographic studies showed that higher parity and larger household sizes are correlated with higher neonatal mortality, but they only reflect patterns of cumulative fertility and constraints on resources, rather than modifiable exposures to risk for neonatal deaths [82, 86]. Both early breastfeeding initiation and antenatal care usage are predictors for service-related variables that coincide with evidence of established maternal and newborn care practice. Due to screening for risk and access to preventable services, early utilization of antenatal care has been associated with positive perinatal results [82, 86, 87]. In addition, initiating breastfeeding in a timely manner is associated with decreased odds of neonatal mortality [88]. In contrast to estimating causal effects, the predictive variables used in this analysis represent characteristics that are used for risk stratification. Biological risk markers—including twin birth, birth size, preceding birth interval, and neonate sex—have been shown to produce well-documented epidemiological associations (e.g., mortality rates for multiple births, low birth weight, short intervals between births, and male sex) [1, 89, 90]. The use of biological risk markers will improve the ability to distinguish high-risk individuals for predictive purposes, but they may not create options for actions to reduce that high risk. In summary, the model provides variables that can be associated with high risk from a predictive perspective; however, the use of these data for planning future interventions will require additional analyses of causation, assessment of feasibility, and evaluation of the health care system.

Weighted LightGBM model can provide a solid foundation for developing evidence-based decision-making related to surveillance of neonatal health in low-resource environments, such as risk stratification and program targeting. However, there are many practical limitations to implementing predictive surveillance frameworks in low-resource environments. Inconsistencies in data quality due to incomplete reporting, recall bias, and delays in data entry may compromise predictive accuracy. There are also significant infrastructure constraints that limit real-world use, such as limited computational resources, poor connectivity, and too few trained personnel. In addition, any predictive model will need to be fully integrated into existing health surveillance systems to be useful, to avoid duplicating efforts, and to ensure the generation of actionable insights. As a result, predictive models will need to have their performance validated prospectively, assessed for operational feasibility, and continuously monitored and evaluated (O&M) to ensure that they can be used reliably in the field [91, 92].

## Conclusion

This study employed multiple machine learning models to evaluate their performance through two evaluation methods, which include stratified five-fold cross-validation and an 80%-20% holdout test that uses different class balancing methods to predict neonatal mortality based on EDHS data. The results indicate that ensemble models, particularly boosting algorithms, consistently outperform other models across various validation and balancing strategies. Thus, boosting algorithms are appropriate because of their robustness and stability for neonatal mortality prediction under conditions of class imbalance.

XGBoost and CatBoost models are robust and stable; however, LightGBM provides the highest recall with a slight F1-score, PR-AUC, or precision tradeoff. The weighted LightGBM model not only had better sensitivity with competitive AUC-PR results but also showed reliable interpretability using SHAP analyses, unlike SMOTENC-based models, which introduce artificial data; therefore, it was the preferred model. XGBoost (unbalanced) and CatBoost (unbalanced) can be alternative prediction models. The use of class weighting was also seen to positively influence sensitivity, with a slight impact on precision and/or overall discriminatory performance, indicating that predictions are generalized with less loss of stability.

The SHAP dependence plot findings reveal that breastfeeding initiation, total number of living children, ANC visits, household size, parity, sex, year, birth size, twin birth, and preceding birth interval are the ten most significant predictors of neonatal mortality. Additionally, the preventative predictive associations demonstrated for timely immediate initiation of breastfeeding and adequate ANC visits were in line with the DHS Program recommendations. The study finds that twins, small birth size, male sex, and zero number of living children exhibited increases risk predicting patterns that matched previously established demographic and clinical risk pathways. This model also identified both nonlinear and threshold relationships regarding parity, household size, and temporal patterns, further establishing the model’s robustness as well as epidemiological validity.

Overall, this study indicates that the interaction patterns found within the weighted LightGBM model identify clinically meaningful risk gradients associated with the prediction of neonatal mortality and provide a solid foundation for developing evidence-based decision-making related to surveillance of neonatal health in low-resource environments, such as risk stratification and program targeting. Future research should assess alternative encoding techniques and conduct an external validation of the model to enhance the generalizability of the findings and establish causation, which will lead to better policy recommendations.

### Limitations

Firstly, estimates of neonatal mortality using data from the EDHS may have been subject to recall and reporting bias that is characteristic of retrospective surveys. Recall bias can result in age heaping and misclassification of deaths that occur near the neonatal–post-neonatal boundary; additionally, very early neonatal deaths may not have been included at all.

Secondly, neonatal death classification relied on reported age at death in EDHS, which may be subject to recall errors and limited misclassification around the 28-day boundary. This misclassification problem creates label noise, which can impact predictive performance, especially for late neonatal death cases.

Thirdly, EDHS does not account for neonatal deaths from maternal mortality due to having only interviewed living women, which creates a survivorship bias. Hence, it eliminates a subset of high-risk neonatal deaths from the training data, which may distort feature importance and limit generalizability to settings where maternal mortality is high.

Fourthly, by combining data from all the EDHS (2000 to 2019), the data set increases sample sizes and statistical stability, but it could also introduce possible temporal dependence. Standard cross-validation assumes that observations are exchangeable; however, there are several possible reasons (e.g., secular trends) that may create structured differences between successive years’ surveys due to changes in the access to health care services, changes in reporting practices, and different socio-economic conditions over time. Since one or more previous years’ observations could potentially be in different folds during cross-validation, the performance of individual folds may be overly optimistic relative to a strict forward-in-time prediction. In the future, researchers should use time-stratified data to validate the results of their work and better estimate temporal generalizability.

## Electronic Supplementary Material

Below is the link to the electronic supplementary material.

Supplementary Material 1

Supplementary Material 2

Supplementary Material 3

## Data Availability

The data used in this study is available in the publicly available domain. The database can be found here: [https://dhsprogram.com/data/available-datasets.cfm].

## Abbreviations

ANC: Antenatal Care
CatBoost: Categorical Gradient Boosting
CV: Cross Validation
DT: Decision Tree
EDHS: Ethiopian Demographic Health Survey
GBM: Gradient Boosting Machine
KNN: K-Nearest Neighbors
LightGBM: Light Gradient Boosting Machine
LR: Logistic Regression
ML: Machine learning
PR-AUC: Precision–Recall Area Under the Curve
RF: Random Forest
SHAP: SHapley Additive exPlanations
SMOTENC: Synthetic Minority Over-sampling Technique for Nominal and Continuous features
XAI: Explainable Artificial Intelligence
XGBoost: Extreme Gradient Boosting
