# Supplementary material for: Interpretable prediction of neonatal mortality and its key predictors using machine learning and SHAP analysis
Source: BMC Med Inform Decis Mak. 2026 May 21;26:266. doi: 10.1186/s12911-026-03567-1 (PMC13371363; doi:10.1186/s12911-026-03567-1)
Supplement: Supplementary file 1 — Supplementary Material 1 [file 12911_2026_3567_MOESM1_ESM.docx]

# **Supplementary file S1**

Table 1. Descriptive Statistics for Categorical Variables with Definitions

| **Variable** | **Variable Definition** | **Category** | **Count** | **Percentage (%)** |
| --- | --- | --- | --- | --- |
| **Mother_Age_Group** | Age of the mother at the time of survey (years) | 25–29 | 1507 | 28.21 |
|  |  | 20–24 | 1173 | 21.95 |
|  |  | 30–34 | 1040 | 19.46 |
|  |  | 35–39 | 796 | 14.90 |
|  |  | 40–44 | 377 | 7.06 |
|  |  | 15–19 | 297 | 5.56 |
|  |  | 45–49 | 153 | 2.86 |
| **Mother_Edu_level** | Highest educational attainment of the mother | No education | 3868 | 72.39 |
|  |  | Primary | 1158 | 21.67 |
|  |  | Secondary | 244 | 4.57 |
|  |  | Higher | 73 | 1.37 |
| **Mom_Age_at_First_Birth** | Mother’s age at first childbirth (years) | 15–17 | 1776 | 33.24 |
|  |  | 20–24 | 1544 | 28.90 |
|  |  | 18–19 | 1196 | 22.38 |
|  |  | <15 | 439 | 8.22 |
|  |  | 25+ | 388 | 7.26 |
| **Marital_Status** | Current marital status of the mother | Married | 4945 | 92.55 |
|  |  | Not Married | 398 | 7.45 |
| **Religion** | Religious affiliation of the mother | Muslim | 2329 | 43.59 |
|  |  | Orthodox | 1954 | 36.57 |
|  |  | Protestant | 882 | 16.51 |
|  |  | Traditional | 98 | 1.83 |
|  |  | Catholic | 40 | 0.75 |
|  |  | Other | 40 | 0.75 |
| **Tetanus_Dose_Preg** | Tetanus toxoid vaccination status during pregnancy | No Vaccine | 4058 | 75.95 |
|  |  | Protective Vaccine | 1032 | 19.31 |
|  |  | Limited Vaccine | 253 | 4.74 |
| **ANC_Visits** | Number of antenatal care visits during pregnancy | No ANC | 3278 | 61.35 |
|  |  | Limited ANC | 1153 | 21.58 |
|  |  | Adequate ANC | 713 | 13.34 |
|  |  | High ANC | 199 | 3.72 |
| **Wealth_Index** | Household wealth status (DHS wealth quintile) | Poorest | 1507 | 28.21 |
|  |  | Poorer | 993 | 18.59 |
|  |  | Middle | 961 | 17.99 |
|  |  | Richer | 917 | 17.16 |
|  |  | Richest | 965 | 18.06 |
| **Drinking_Water_Type** | Type of household drinking water source | Unimproved | 2728 | 51.06 |
|  |  | Improved | 2615 | 48.94 |
| **Twin** | Whether the birth was a multiple birth | No | 5058 | 94.67 |
|  |  | Yes | 285 | 5.33 |
| **Birth_Size** | Mother’s reported size of child at birth | Large | 1830 | 34.25 |
|  |  | Average | 1785 | 33.41 |
|  |  | Small | 1728 | 32.34 |
| **Preceding_Birth_Interval_Categorized** | Time interval between consecutive births | Recommended | 2607 | 48.79 |
|  |  | First Birth | 1158 | 21.67 |
|  |  | Short | 578 | 10.82 |
|  |  | Very Short | 514 | 9.62 |
|  |  | Long | 486 | 9.10 |
| **Breastfeeding_Initiation_Category** | Timing of breastfeeding initiation after birth | Immediate | 3243 | 60.70 |
|  |  | Delayed | 1748 | 32.72 |
|  |  | Early | 352 | 6.59 |
| **Sex_of_Child** | Biological sex of the child | Male | 2859 | 53.51 |
|  |  | Female | 2484 | 46.49 |
| **Region** | Administrative region of residence | Oromia | 954 | 17.86 |
|  |  | Amhara | 810 | 15.16 |
|  |  | SNNP | 660 | 12.35 |
|  |  | Benishangul Gumuz | 558 | 10.44 |
|  |  | Somali | 519 | 9.71 |
|  |  | Tigray | 510 | 9.55 |
|  |  | Gambela | 360 | 6.74 |
|  |  | Afar | 324 | 6.06 |
|  |  | Harari | 291 | 5.45 |
|  |  | Dire Dawa | 216 | 4.04 |
|  |  | Addis Ababa | 141 | 2.64 |
| **Residence_Type** | Place of residence | Rural | 4537 | 84.91 |
|  |  | Urban | 806 | 15.09 |
| **Climate_Zone** | Agro-ecological zone of residence | Weyna Dega | 2487 | 46.55 |
|  |  | Kolla | 1820 | 34.06 |
|  |  | Dega | 856 | 16.02 |
|  |  | Wurch | 180 | 3.37 |
| **Delivery_Facility** | Place of delivery | Home | 4316 | 80.78 |
|  |  | Health Facility | 471 | 8.82 |
|  |  | Hospital | 464 | 8.68 |
|  |  | Other | 92 | 1.72 |
| **Contraceptive_Method** | Current contraceptive method use | Not using | 4384 | 82.05 |
|  |  | Modern | 904 | 16.92 |
|  |  | Traditional | 55 | 1.03 |
| **Neonate_Dead** | Neonatal mortality outcome (death within 28 days of birth) | No | 3562 | 66.67 |
|  |  | Yes | 1781 | 33.33 |

Table 2. Summary Table for Numerical Variables with Definitions

| **Variable** | **Variable Definition** | **Mean** | **Std Dev** | **Median** |
| --- | --- | --- | --- | --- |
| **HH_Size** | Total number of household members living in the household | 5.854 | 2.392 | 6 |
| **Total_Children_Ever_Born** | Total number of children ever born to the mother (lifetime fertility) | 4.378 | 2.648 | 4 |
| **Number_of_Living_Children** | Number of the mother’s children who are currently alive | 1.558 | 1.380 | 1 |

Table 3. Summary Table for temporal variable

| **Year** | **Count** | **Percentage (%)** |
| --- | --- | --- |
| 2000 | 1488 | 27.85 |
| 2005 | 1026 | 19.2 |
| 2011 | 1245 | 23.3 |
| 2016 | 960 | 17.97 |
| 2019 | 624 | 11.68 |

Table 4. Optimized hyperparameter settings

| **Model** | **Parameters tuned in SMOTENC Balanced Learning** |
| --- | --- |
| **LR** | C=0.140585, penalty='l2', solver='liblinear', max_iter=2000 |
| **KNN** | n_neighbors=13, weights='distance', algorithm='auto', leaf_size=30, p=2 |
| **DT** | max_depth=7, min_samples_split=5, min_samples_leaf=3, random_state=42 |
| **RF** | max_depth=17, n_estimators=231, min_samples_split=2, min_samples_leaf=1, random_state=42 |
| **GBM** | max_depth=3, n_estimators=383, learning_rate=0.02231451, subsample=1.0, random_state=42 |
| **XGBoost** | max_depth=4, n_estimators=261, learning_rate=0.026367631, eval_metric='logloss', subsample=1.0, colsample_bytree=1.0, use_label_encoder=False, random_state=42 |
| **LightGBM** | max_depth=5, n_estimators=190, learning_rate=0.02243626, num_leaves=31, subsample=1.0, random_state=42 |
| **CatBoost** | n_estimators=248, learning_rate=0.022025866, depth=8, l2_leaf_reg=3, border_count=32, random_state=42, silent=True |
