# Supplementary material for: Interpretable prediction of neonatal mortality and its key predictors using machine learning and SHAP analysis
Source: BMC Med Inform Decis Mak. 2026 May 21;26:266. doi: 10.1186/s12911-026-03567-1 (PMC13371363; doi:10.1186/s12911-026-03567-1)
Supplement: Supplementary file 2 — Supplementary Material 2 [file 12911_2026_3567_MOESM2_ESM.docx]

# **Supplementary file S3**

Table 1. Feature importance value

| **Feature** | **MeanAbsSHAP** |
| --- | --- |
| Breastfeeding_Initiation_Category | 1.61 |
| Number_of_Living_Children | 0.37 |
| ANC_Visits | 0.36 |
| Total_Children_Ever_Born | 0.27 |
| HH_Size | 0.25 |
| Sex_of_Child | 0.23 |
| Survey_Year_GC | 0.15 |
| Birth_Size | 0.15 |
| Tetanus_Dose_Preg | 0.12 |
| Twin | 0.11 |


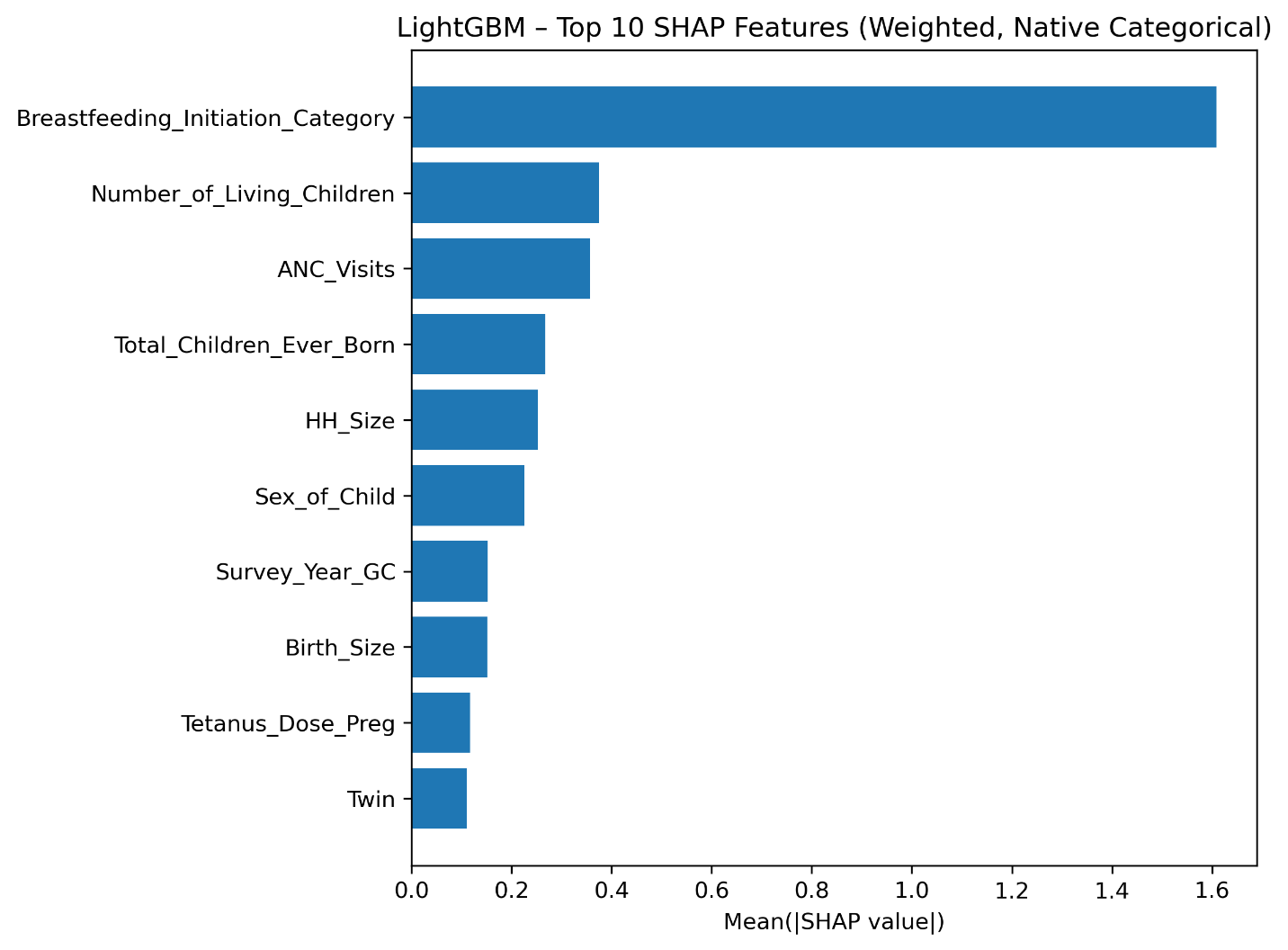


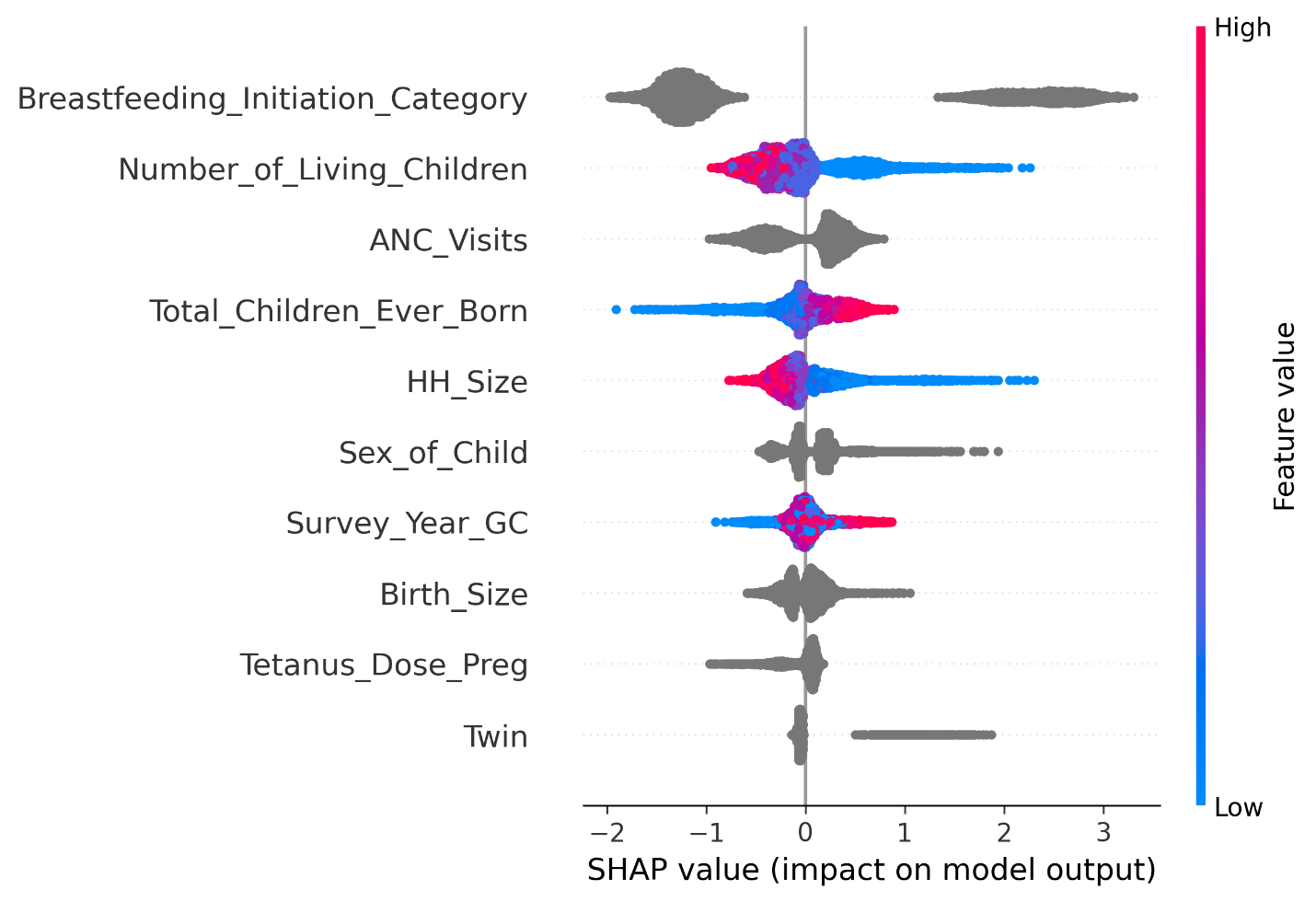


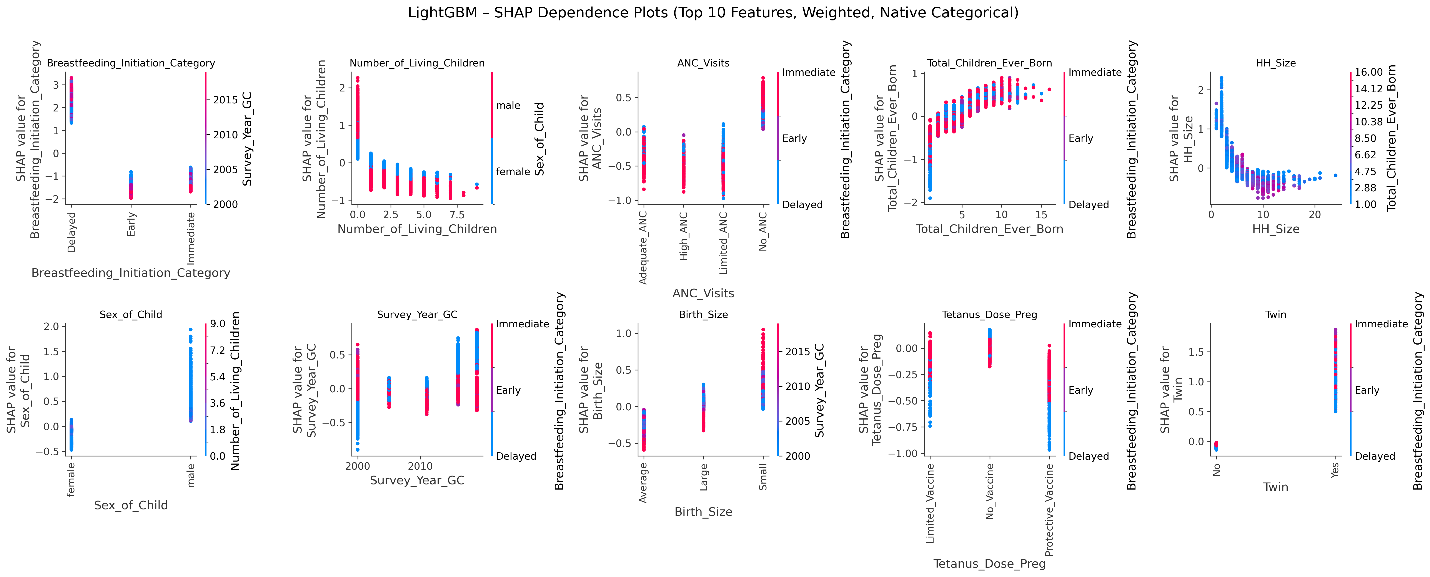


Figure 1. SHAP summary plots for top ten features of weighted LightGBM with native categorical encoding evaluated using five-fold CV: A) Feature importance bar plot, B) Beeswarm plot C) Pairwise dependent plot
