# Supplementary material for: Interpretable prediction of neonatal mortality and its key predictors using machine learning and SHAP analysis
Source: BMC Med Inform Decis Mak. 2026 May 21;26:266. doi: 10.1186/s12911-026-03567-1 (PMC13371363; doi:10.1186/s12911-026-03567-1)
Supplement: Supplementary file 3 — Supplementary Material 3 [file 12911_2026_3567_MOESM3_ESM.docx]

# **Supplementary file S2**


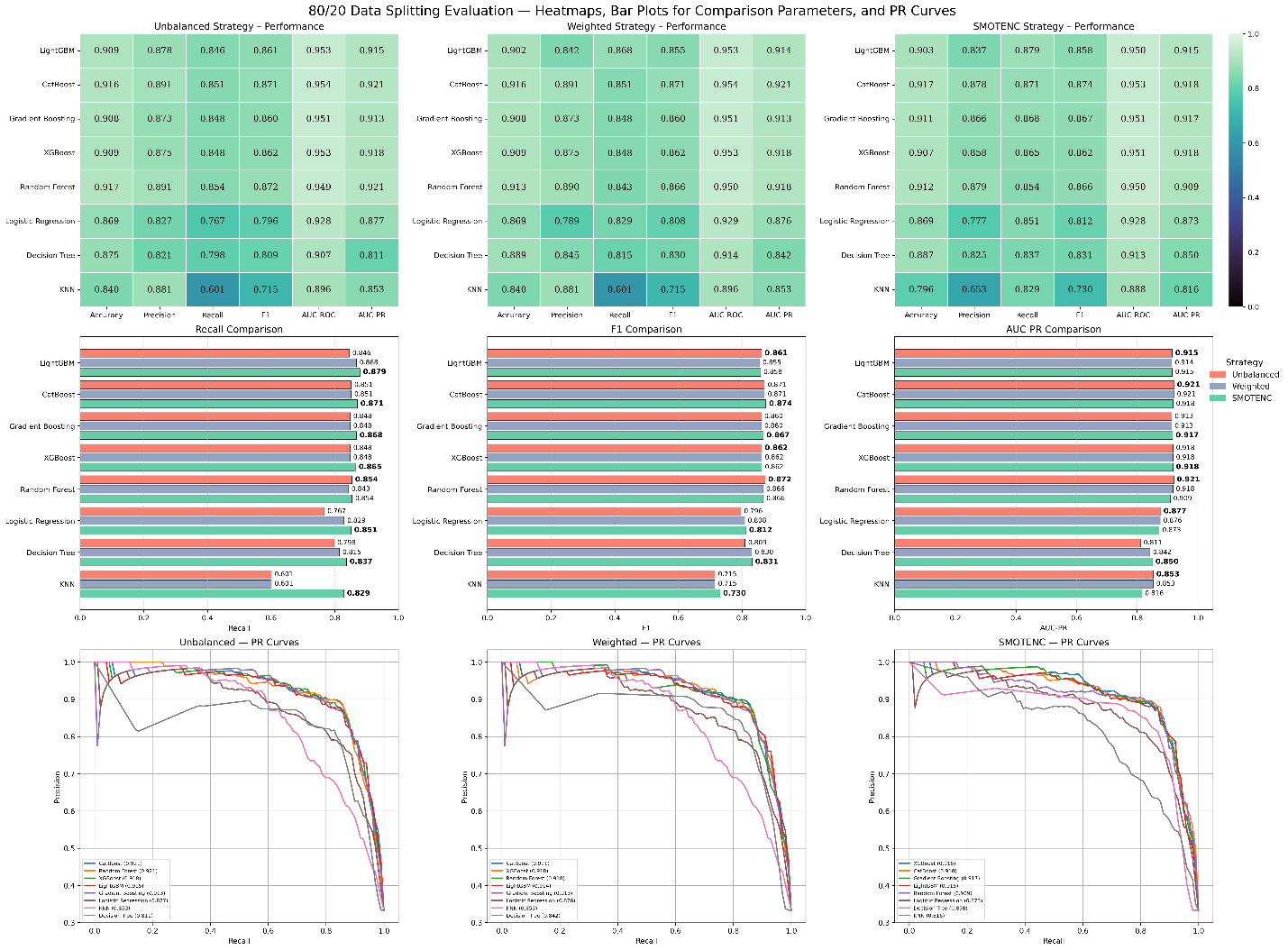


Figure 1. The 80/20 hold-out validation performance evaluations of models across class balancing strategies; A) Heatmap plots, B) Comparison bar plots, and C) PR-AUC plots


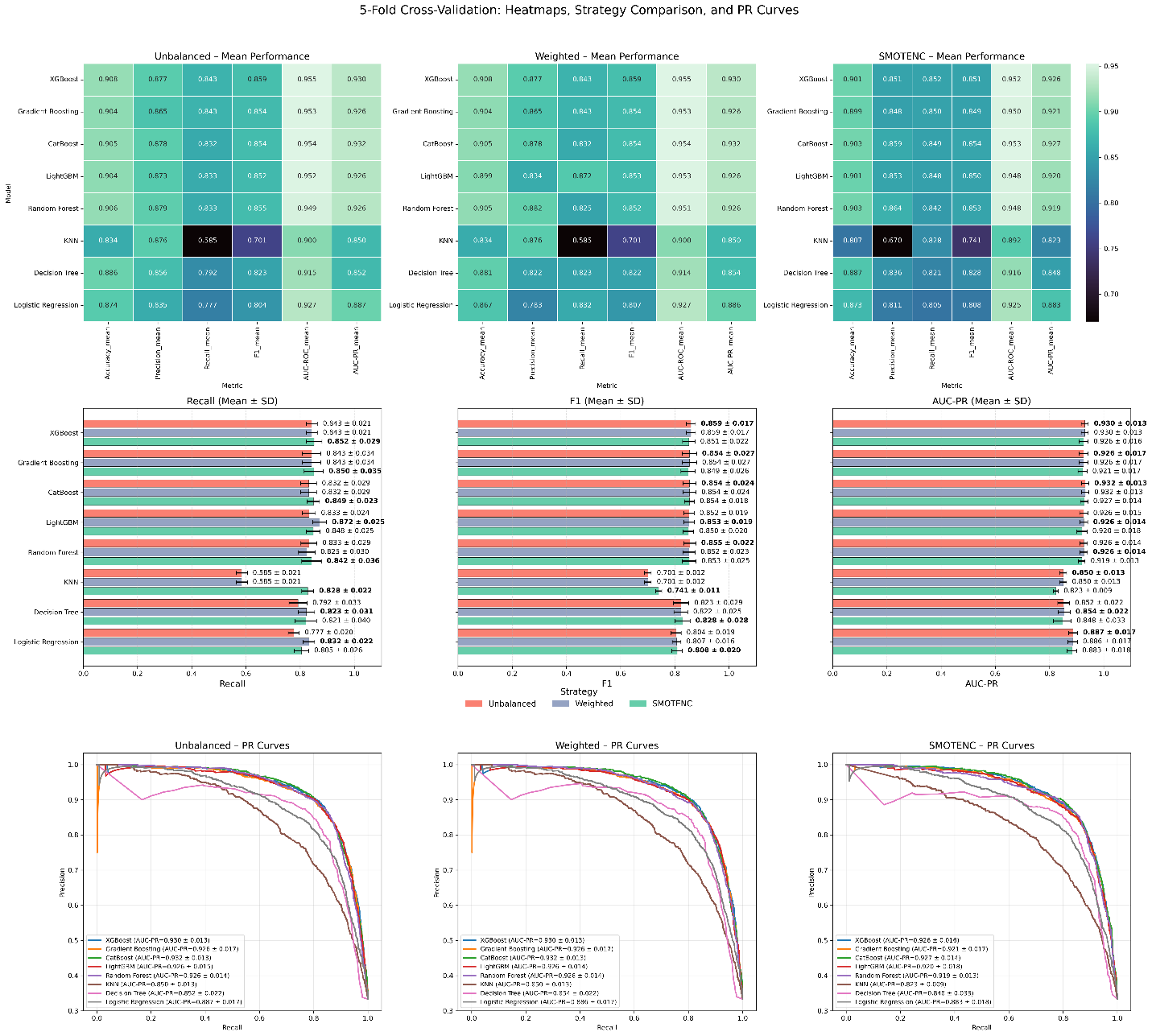


Figure 2. The five-fold CV performance evaluations of models across class balancing strategies; A) Heatmap plots, B) Comparison bar plots, and C) PR-AUC plots


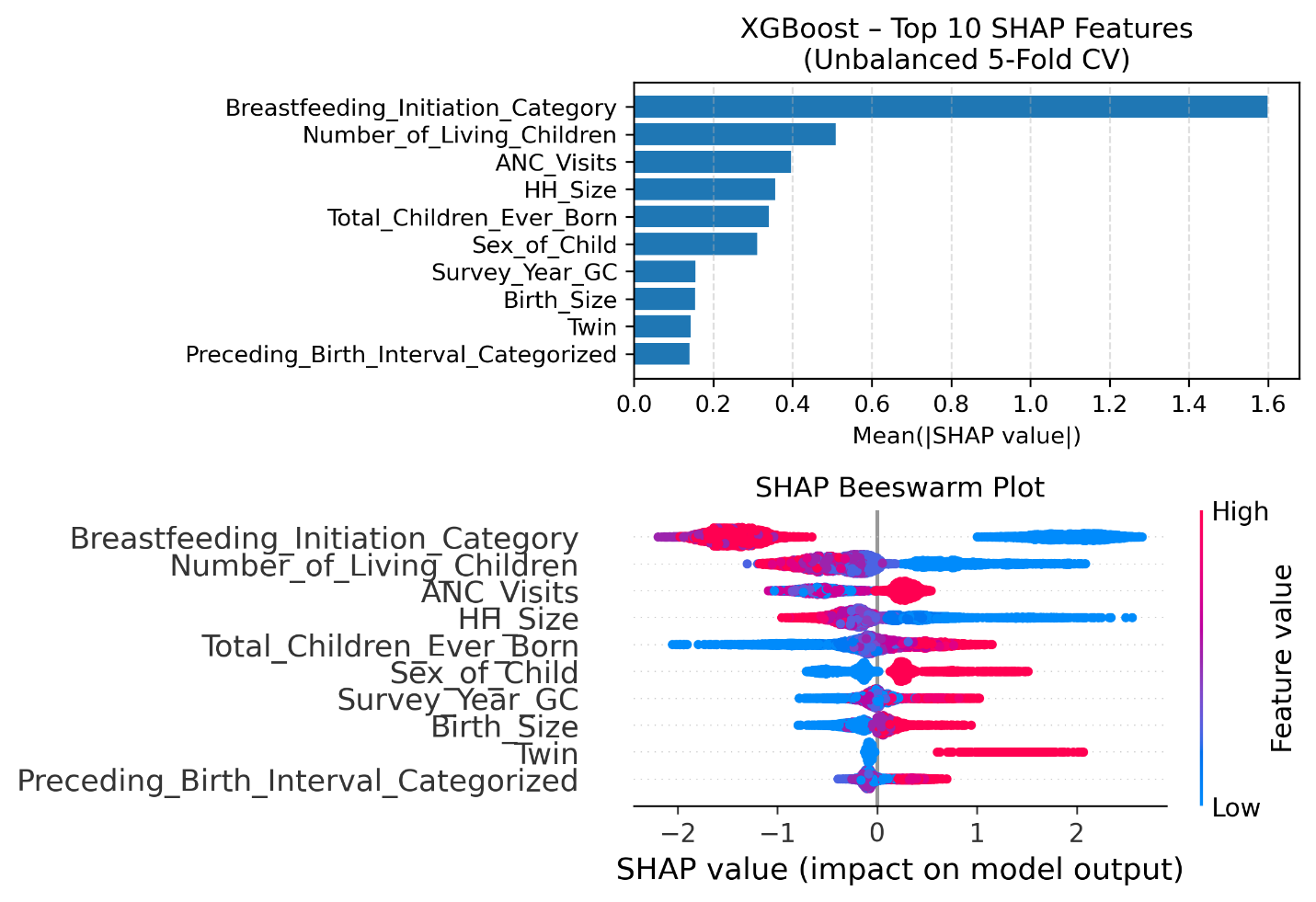


Figure 3. SHAP summary plot for top ten features of unbalanced XGBoost model evaluated using stratified five-fold CV: A) Feature importance bar plot, B) Beeswarm plot


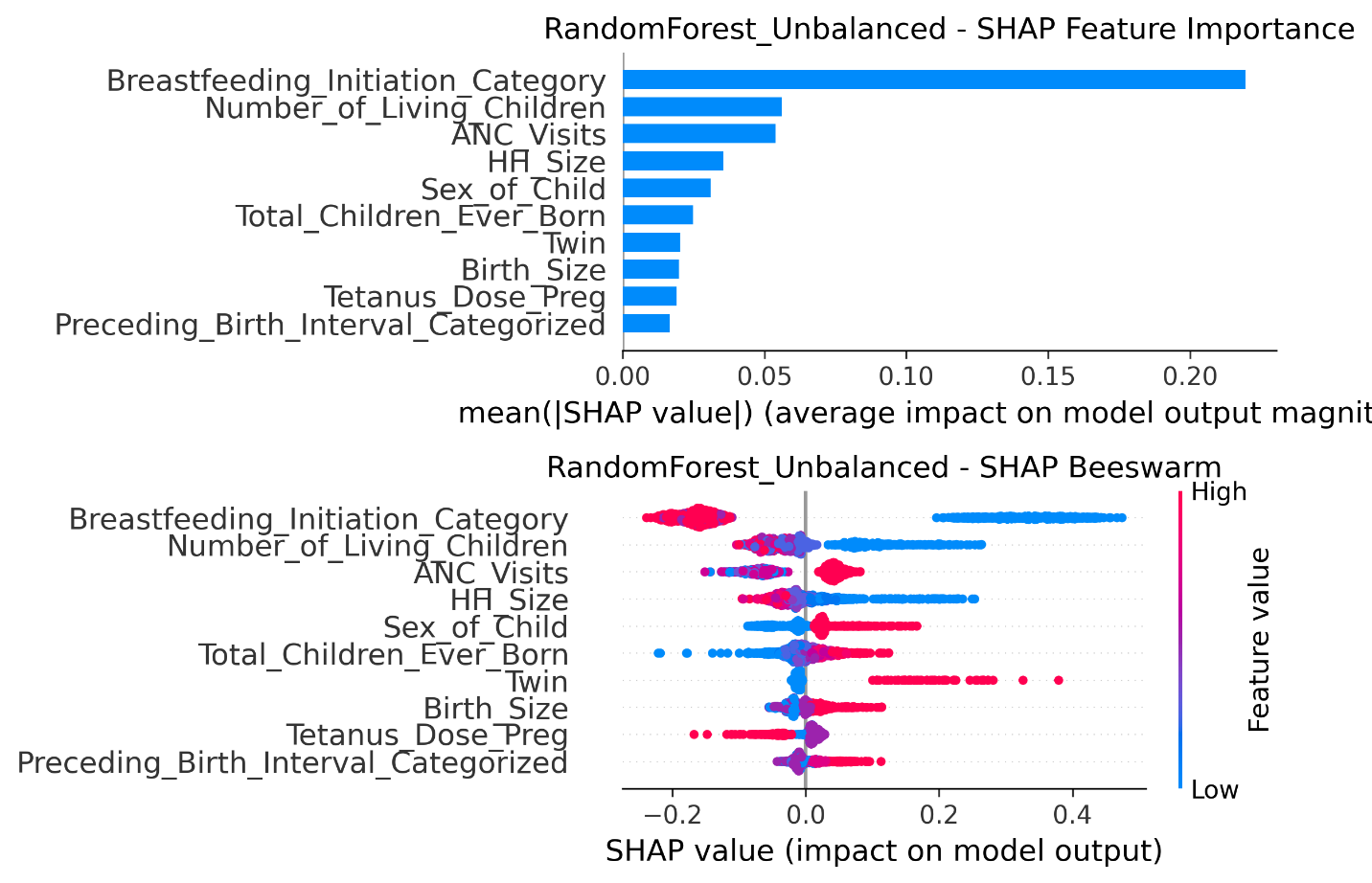


Figure 4. SHAP summary plots for top ten features of unbalanced Random Forest evaluated using 80/20 hold-out validation: A) Feature importance bar plot, B) Beeswarm plot


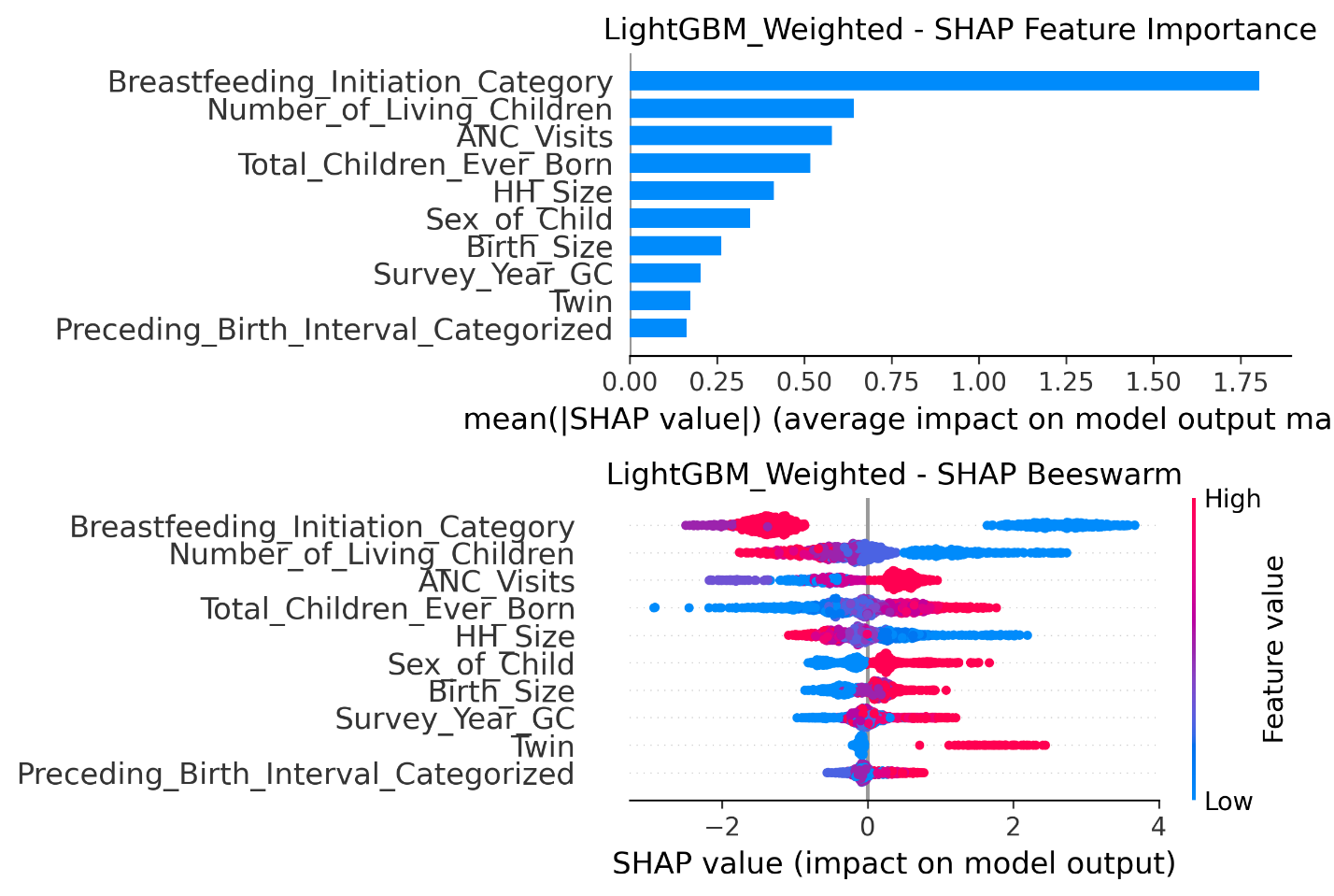


Figure 5. SHAP summary plots for top ten features of weighted LightGBM evaluated using 80/20 hold-out validation: A) Feature importance bar plot, B) Beeswarm plot


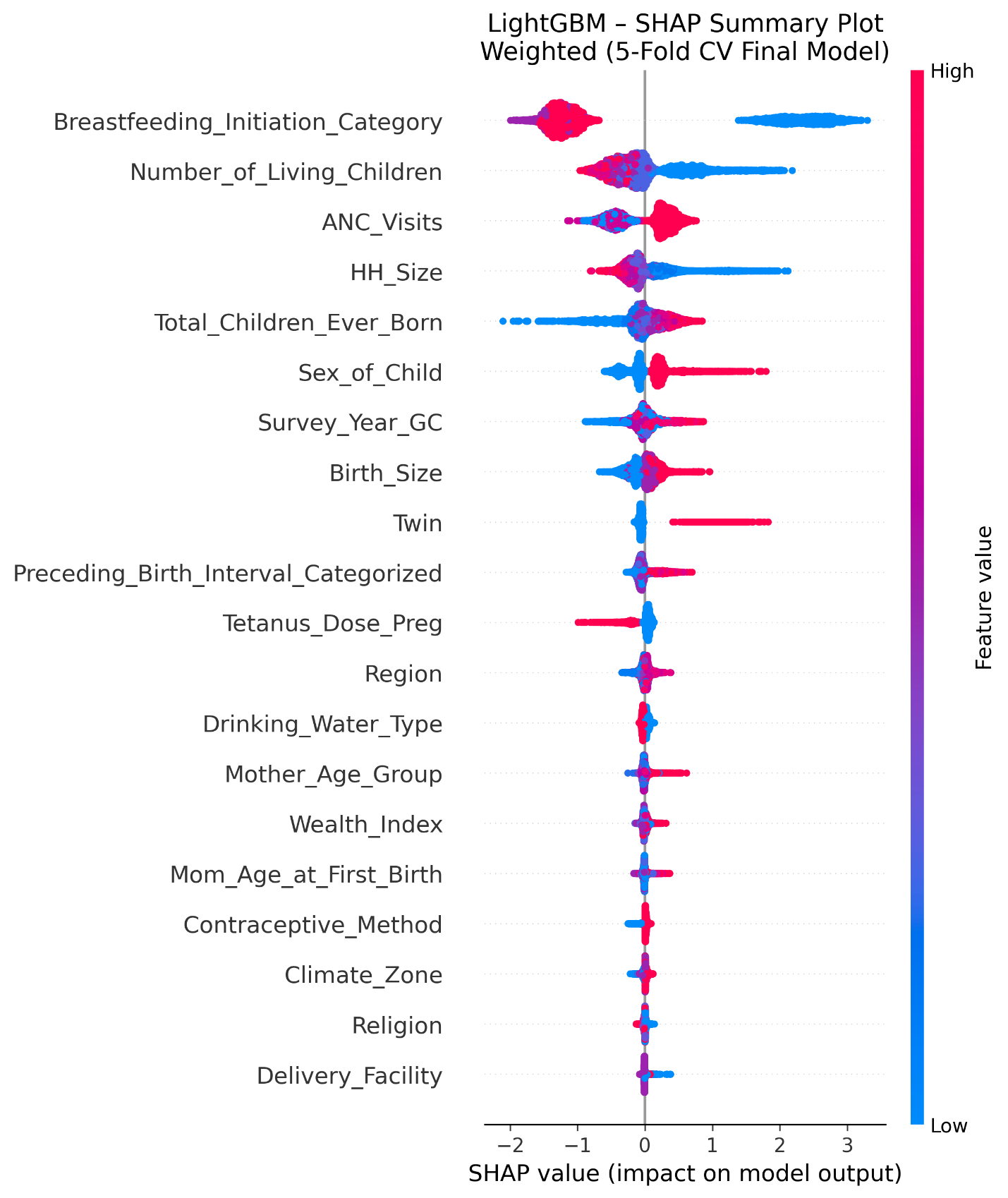


Figure 6. SHAP Beeswarm plots for top 20 features of weighted LightGBM evaluated using five-fold CV
